# Robotic-assisted total knee arthroplasty is more advantageous for knees with severe deformity: a randomized controlled trial study design

**DOI:** 10.1097/JS9.0000000000000002

**Published:** 2023-03-24

**Authors:** Run Tian, Xudong Duan, Ning Kong, Xinhua Li, Jian Wang, Hua Tian, Zhanjun Shi, Shigui Yan, Jingyi Lyu, Kunzheng Wang, Pei Yang

**Affiliations:** Departments of a Bone and Joint Surgery; b Radiology, The Second Affiliated Hospital of Xi’an Jiaotong University, Xian; c Department of Orthopedics, Nan Fang Hospital of Nan Fang Medical University, Guangzhou; d Department of Orthopedics, Third Hospital of Peking University, Beijing; e Department of Bone and Joint, the Second Affiliated Hospital Zhejiang University School of Medicine, Hangzhou; f Hang Zhou JianJia Robot Co. Ltd, Hangzhou, China

**Keywords:** lower extremity alignment deviations, robotic-assisted, total knee arthroplasty

## Abstract

A prospective, multicenter, randomized controlled trial was conducted to explore the short-term effect of a new robotic-assisted total knee arthroplasty (TKA) system, and the clinical and radiographic effectiveness between the robotic-assisted system and conventional TKA were compared and analyzed.

**Materials and Methods:**

Overall, 144 patients were randomly divided into two groups, wherein 72 patients underwent TKA using the robotic‑assisted system and 72 underwent conventional TKA. The demographic data and radiographic parameters of the patients were collected. The factors influencing postoperative hip–knee–ankle (HKA) angle deviation were determined by multiple linear regression. Clinical outcomes including postoperative Knee Society score, 10-cm visual analog scale, and range of motion (ROM) and radiographic results including the deviation value of coronal tibial component angle, coronal femoral component angle (CFCA), sagittal tibial component angle, sagittal femoral component angle (SFCA), and HKA angle as well as the rate of outliers in each angle were observed and compared between the two groups.

**Results:**

The preoperative demographic data and imaging parameters, including Knee Society score, ROM, sex, surgical side, age, BMI, preoperative HKA angle, preoperative HKA angle deviation, and visual analog scale, showed no significant differences between groups. The robotic‑assisted system group (RAS group) showed a postoperative malalignment of 3.2% for a mechanical axis higher than 3° and the conventional techniques group (CON group) showed a postoperative malalignment of 41.0% for a mechanical axis higher than 3°; the difference was statistically significant (*P*<0.001). According to the results of multiple linear regression analysis, when the preoperative HKA angle deviation increased by 1°, the postoperative HKA angle deviation increased by 0.134° (*β*=0.134 min; 95% CI: 0.045–0.222). Therefore, patients were divided into a slight lower extremity alignment deviation group (preoperative HKA angle deviation <6°) and severe lower extremity alignment deviation group (preoperative HKA angle deviation ≥6°). For the patients with preoperatively slight lower extremity alignment deviation, the rate of postoperative HKA angle outlier in the RAS group was better than that in the CON group, and the operation duration in the RAS group was significantly longer than that in the CON group (*P*<0.05). In the patients with a preoperative HKA angle deviation ≥6°, the rate of postoperative HKA angle and CFCA outliers in the RAS group was better than that in the CON group; the operation duration in the RAS group was significantly longer than that in the CON group, and the HKA angle deviation and CFCA deviation in the RAS group were significantly lower than those in the CON group (*P*<0.05). No significant difference was observed in other indexes between the two groups (*P*>0.05).

**Conclusion:**

This new robotic-assisted TKA system is safe and effective. The authors found that preoperative HKA angle deviation affects the postoperative HKA angle deviation. The robotic-assisted system has similar results to those reported by the traditional method with regard to restoring the mechanical axis of the leg and improving prosthesis alignment and clinical outcomes in patients with slight lower extremity alignment deviations preoperatively. For patients with severe preoperative lower extremity alignment deviations, the effectiveness in terms of the improvement in mechanical axis of the leg and prosthesis alignment were better with the robotic-assisted system, whereas the effectiveness of clinical outcomes was similar. A larger sample size and longer follow-up period are needed to determine whether the improved mechanical axis of the leg and prosthesis alignment observed with the robotic-assisted system can achieve better long-term radiographic and clinical outcomes.

## Introduction

Total knee arthroplasty (TKA) is an effective treatment for various advanced knee diseases with good clinical outcomes^1^. The main purpose of TKA currently is to restore neutral mechanical axis in patients^2^. Although the surgical technique of TKA is becoming increasingly mature in recent years, some problems, such as imprecise placement of prosthesis and poor recovery of mechanical axis still remain, leading to patient dissatisfaction with the operation, increased polyethylene wear, and premature loosening consequently leading to early revision surgery^3^. Thus, accurate placement of the prosthesis and normal alignment of the lower limb axis play a key role in reducing postoperative complications and improving the survivor time of the prosthesis, and directly affects long-term TKA results^4^. In conventional surgical techniques in TKA, surgical instruments are matched to determine the placement of prosthesis, which is highly dependent on the operator’s experience, and has some problems such as low accuracy and repeatability^5^. To address these problems, a robotic‑assisted system is applied in TKA. It is considered that the robotic‑assisted system can effectively improve the accuracy of prosthesis placement and theoretically improve the limb axis alignment^6^. However, current research is controversial^7^–^9^. Some studies believe that a robotic‑assisted system cannot achieve better surgical outcomes than conventional TKA techniques^10^, but in another study, Liow *et al*.^11^ reported that a robotic‑assisted system can significantly improve implant alignment in TKA. The reason behind these contradictory results still needs to be further explored.

**Figure 1:**
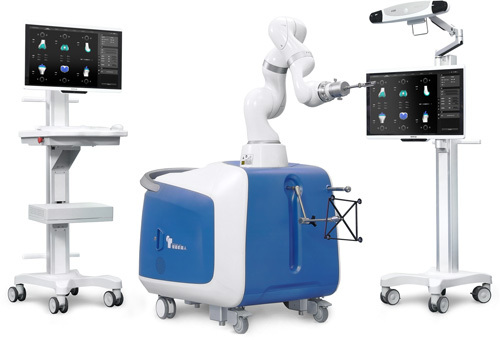
The robot console and the surgical platform of the Jianjia robot-assisted total knee arthroplasty system.

To date, only few robotic-assisted TKA systems have been approved by the National Medical Products Administration of China^12^. A new robotic-assisted TKA system (Hangzhou Jianjia Robot Co. Ltd., Jianjia, China) has recently been designed (Fig. 1). This arthroplasty system used a seven-axis robotic arm, in theory more similar to the characteristics of movement of a unilateral arm of the human body than other systems. The robotic arm provided a large degree of movement capability without any reduction in stability. In addition, this system is compact and flexible in terms of volume, occupying only a small space in the operating room, but overcomes obstacles more easily in narrow spaces than other robotic-assisted TKA systems on the market because the design of the human–computer interaction strategy, which was optimized for the needs and surgical habits of Chinese doctors. In the present study, we conducted a multicenter, prospective, randomized controlled study to verify the effectiveness and safety of this system in humans. We discussed the factors affecting postoperative HKA angle deviation, which was considered as the main evaluation index, and compared and analyzed the imaging and clinical effectiveness between the abovementioned conventional TKA and TKA using the robotic‑assisted system. Further, we performed stratified analysis of patients with different degrees of preoperative lower extremity deformity and attempted to determine why the robotic-assisted knee replacement system showed conflicting results in different studies.

## Highlights

- We verify the effectiveness and safety of a new robotic-assisted total knee arthroplasty (TKA) system (Jianjia, Hangzhou Jianjia Robot Co. Ltd.) in humans.
- We discussed the factors affecting postoperative hip–knee–ankle (HKA) angle deviation, which was considered as the main evaluation index.
- We performed stratified analysis of patients with different degrees of preoperative lower extremity deformity and attempted to determine why the robotic-assisted knee replacement system showed conflicting results in different studies.

## Materials and methods

### Study design and patients

This multicenter, prospective, randomized controlled study was conducted between June 2021 and December 2021. Four centers located in different regions of China were included in this study: the Second Affiliated Hospital of Xi’an Jiaotong University, the Second Affiliated Hospital Zhejiang University School of Medicine, the Peking University Third Hospital, and the Nanfang Hospital Southern Medical University. The study was approved by the Ethics Committee of the second affiliated Hospital of Xi’an Jiaotong University (Permit Number: XJTU 2021-015). The trial is designed as a randomized controlled study (Trial Number, ChiCTR2200065786). The study protocol and ethics approval are available on this website (http://www.chictr.org.cn/showproj.aspx?proj=168010).This study was presented according to the CONSORT guidelines^13^. All operations were performed by experienced doctors, and an informed consent form was obtained from each patient. Patients aged between 18 and 85 years old who planned to undergo their first unilateral TKA operation were included. The exclusion criteria were as follows: BMI >35 kg/m^2^; acute or chronic infection of the proposed operation site or systemic infection; severe osteoporosis; serious systemic diseases that could make the patient unsuitable for the operation; allergy to implant materials; serious mental illness; lower limb neuromuscular diseases and other diseases that affect normal functional rehabilitation after operation; hip disease with obvious bone defect or significant limitation of joint movement on the side to be operated.

### Randomization

**Figure 2:**
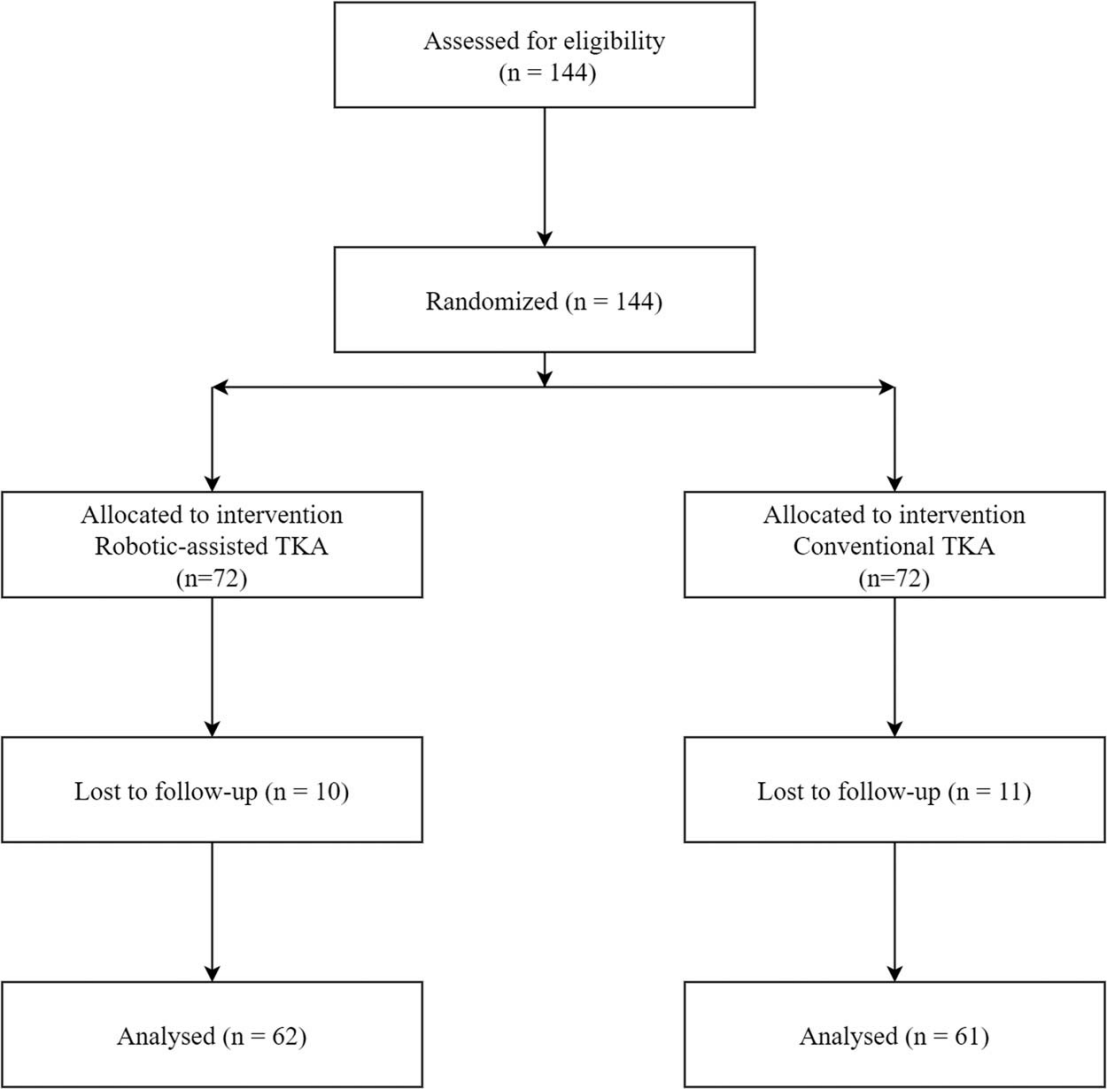
A consolidated standard of reporting trials flow diagram is shown. In all, 62 patients were treated with robotic-assisted total knee arthroplasty (TKA) and 61 were treated with conventional TKA.

Each center competed for enrollment, adopted the block random method, used an interactive web response system to assign patients who agreed to participate in the study to either the conventional technique (CON) group or the robotic‑assisted system (RAS) group in a 1 : 1 ratio, and then introduced the results into the central randomized system. The researchers intervened patients through the results of the randomized allocation (Fig. 2).

### Sample size calculation

According to the relevant literature and considering clinical practice factors, the deviation rate of the lower limb mechanical axis in the RAS group is expected to be 8%, and that in the CON group is expected to be 28%, with the critical value of clinical excellence being 0%^5^,^14^. The setting parameters were as follows: *α*=0.025 (unilateral), *β*=0.2, the ratio of RAS group to CON group was 1 : 1. The sample size estimation formula for excellent clinical verification was used, and the results showed that 57 patients were needed in each group. Considering that about 20% of the cases may have lost to follow-up during the study, a total of 72 patients would be needed in each group.

### Interventions

Based on the estimated results, we included 144 patients who underwent TKA. The patients were randomly divided into two groups: the CON group (72 patients) included patients who underwent conventional TKA, with the operation process performed as described in the technique manual provided by the manufacturer; the RAS group (72 patients) included patients who underwent robotic-assisted knee arthroplasty using the robotic-assisted system developed by Hangzhou Jianjia Robot Co. Ltd. This robot is a semiactive surgical robot, which can generate a 3D bone model based on the patient’s preoperative computed tomography scan, help doctors determine the most appropriate prosthesis model and size before operation, and improve prosthesis placement. The same prosthesis (Zimmer Biomet, Warsaw, Indiana, USA) was used in all surgeries. The patient was operated under general anesthesia unless there was a clear contraindication. A lower limb tourniquet was used in all patients, and the tourniquet pressure was 300 mm Hg. The anterior median approach was followed in all surgeries, and the incision was ~14 cm long.

**Figure 3:**
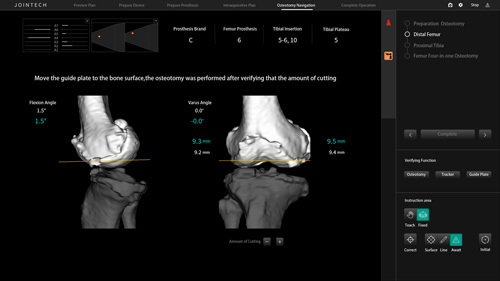
The distal femur osteotomy procedure using the Jianjia robot-assisted total knee arthroplasty system.

**Figure 4:**
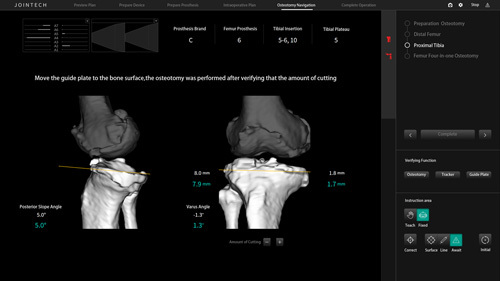
Tibial osteotomy procedure using the Jianjia robot-assisted total knee arthroplasty system.

In the RAS group, when the robotic‑assisted system was applied for TKA, two 3.0 mm steel needles were inserted perpendicular to the femur at ~5 cm above the front articular line of the femur. Following this, the guide plate was fixed and connected with the femoral reflex ball to adjust the position of the receiver and make the receiving signal stable. After the registration of the femur was completed and verified, the robotic arm was positioned, and the cutting guide was inserted. The distal femur cut was performed after verifying that the amount of cutting matched with preoperative planning, and the robotic arm was adjusted again. After determining the osteotomy volume of the anterior and posterior femoral condyles and verifying it again, the robotic arm was positioned and the osteotomy guide was inserted to complete the osteotomy of the femur. Subsequently, two 3.0 mm steel needles were inserted vertically to the tibia at about 5 cm below the joint line of the tibia. After the registration of the tibia was completed and verified by the positioning needle with a reflex ball, the robotic arm was positioned, fixed after the positioning was confirmed, and the cutting guide was inserted to verify that the cutting matched with preoperative planning (Figs 3, 4). In the CON group, the distal femur was resected following the intramedullary technique, and the proximal tibia was resected following the extramedullary technique. All patients were treated with antibiotic bone cement to fix the tibial and femoral prosthesis. The postoperative rehabilitation plan and the mode of postoperative analgesia were the same for both groups.

### Outcome measures

**Figure 5:**
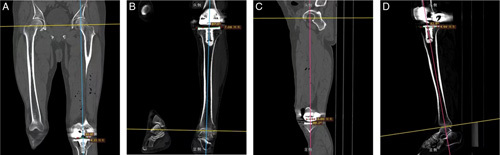
Postoperative computed tomography scans. (a) Measurement of the coronal femoral component angle. (b) Measurement of the coronal tibial component angle. (c) Measurement of the sagittal femoral component angle. (d) Measurement of the sagittal tibial component angle.

Within 7 days after operation, all patients underwent computed tomography and the following angles were measured to evaluate the position of the knee prosthesis: (1) coronal tibial component angle (CTCA, neutral=90°), the angle between the base tangent of the tibial plateau and the medial side of the tibial mechanical axis; (2) coronal femoral component angle (CFCA, neutral=90°), the angle between the base tangent of the femoral prosthesis and the outside of the femoral mechanical axis; (3) sagittal tibial component angle (STCA, neutral=90°), the posterior angle of the tangent line between the base of the tibial plateau and the anatomical axis of the proximal tibia; and (4) sagittal femoral component angle (SFCA, neutral=90°), the posterior angle between the tangent line of the base of the femoral prosthesis and the anatomical axis of the distal femur (Fig. 5). Further, before and 12 weeks after the operation, all patients underwent standing Anterior-Posterior hip-to-ankle radiographs and the hip–knee–ankle angle (HKA angle, neutral=180°), which is the angle between the mechanical axis of the femur and the mechanical axis of the tibia, was measured^15^,^16^. The abovementioned angles were measured by two trained radiologists, and the average values of the measurements were considered. A deviation between each angle and the neutral position of ±3° was considered to be acceptable, and a deviation beyond this range predisposed the patient to early implant failure^17^. After the measurement was completed, the deviation (absolute value) between the measured value and the neutral angle value for HKA, SFCA, STCA, CTCA, and CFCA was calculated. The rate of outliers for each angle in the total was calculated at the same time^18^. Meanwhile, the clinical outcomes of all patients were evaluated by Knee Society score (KSS), a 10-cm visual analog scale (VAS), and range of motion (ROM) before and 12 weeks after operation. Any complications found during the study were recorded.

A total of 123 patients were evaluated based on the clinical and imaging results as 10 patients in the RAS group refused to participate in follow-up evaluation and 11 patients in the CON group refused to participate in the follow-up evaluation.

### Statistical analysis

A Kolmogorov–Smirnov test was used to evaluate the normal distribution of the quantitative data. Normally distributed measurements are presented as mean±SD. Measurements with skewed distributions are presented as medians (interquartile range) and all categorical data as percentages. Levene’s test was used to evaluate the homogeneity of data variance. The significance of normally distributed or approximate normally distributed data was evaluated using a Student *t* test. A Wilcoxon test was used for skewed distribution data, and a *χ* ^2^ test for categorical data. Statistical significance was defined as a *P*<0.05. With the postoperative HKA angle deviation as the dependent variable and the test level *α* set at 0.05, the general demographic data and imaging parameters of the patients were analyzed by single-factor linear regression analysis. If the independent variable showed *P*<0.05 in the single-factor linear regression analysis, the independent variable was included in the multiple linear regression model to determine the variables that are the influencing factors of postoperative HKA angle deviation according to the statistical results. All data were analyzed using SPSS25.0 statistical software (version 25.0; SPSS, New York, NY).

## Results

There was no significant difference in preoperative demographic data and imaging parameters, including KSS, ROM, sex, surgical side, age, BMI, preoperative HKA angle, preoperative HKA angle deviation, and VAS score, between the two groups (Table 1). The RAS group showed postoperative malalignment of 3.2% for mechanical axis higher than 3° and the CON group showed a postoperative malalignment of 41.0% for mechanical axis higher than 3°; the difference was statistically significant (*P*<0.001).

**Table 1:** General parameters of patients

	RAS	CON	Statistic	*P* value
*N*	62	61		
Age	68.17±7.59	68.84±7.12	*t*=−0.508	0.612
Preoperative HKA angle	173.05±6.58	172.03±8.19	*t*=0.766	0.445
Preoperative HKA angle deviation	8.25±4.83	10.06±5.36	*t*=−1.972	0.051
Preoperative VAS	5.97±2.14	6.11±2.30	*t*=−0.347	0.729
Sex				
Male	13	15		
Female	49	46	*χ* ^2^=0.229	0.632
Surgical side				
Left	33	30		
Right	29	31	*χ* ^2^=0.201	0.654
Preoperative KSS	43.03±18.78	40.51±17.51	*t*=0.771	0.442
BMI (kg/m^2^)	25.62±3.08	26.64±3.33	*t*=−1.771	0.079
Preoperative ROM	112.74±20.64	109.77±20.10	*t*=0.809	0.420

CON, conventional techniques; KSS, Knee Society score; RAS, robotic‑assisted system; ROM, range of motion; VAS, 10-cm visual analog scale.

### Complications

In the RAS group, the following complications were reported: one case of surgical incision swelling, five cases of surgical incision oozing blood, and one case of poor incision healing. In the CON group, the following complications were reported: three cases of poor incision healing, five cases of surgical incision oozing blood, one case of venous thrombosis of lower extremities, and two cases of surgical incision swelling. There were no between-group differences in terms of the frequency with which complications occurred (*P*>0.05). No complications that might be specifically related to robotic assistance were observed in the RAS group.

### Factors affecting postoperative HKA angle deviation

To screen for factors affecting postoperative HKA angle, we first subjected the demographic data (age, preoperative KSS, sex, surgical side, and interventions), radiographic parameters (preoperative HKA angle and preoperative HKA angle deviation), and operation duration to single-factor linear regression analysis. The results showed that the preoperative HKA angle (*P*=0.004), preoperative HKA angle deviation (*P*<0.001), preoperative KSS (*P*=0.009), preoperative ROM (*P*=0.016), and interventions (*P*<0.001) may significantly affect the postoperative HKA angle deviation (Table 2).

**Table 2:** Results of single-factor linear regression analysis

Variables	*β*	*β* ate	95%CI	*t*	*P* value
Age	0.045	0.158	−0.006 to 0.095	1.759	0.081
BMI	0.068	0.105	−0.047 to 0.183	1.166	0.246
Sex	0.083	0.017	−0.805 to 0.971	0.185	0.854
Surgical side	−0.286	−0.069	−1.029 to 0.458	−0.761	0.448
Preoperative HKA angle	−0.072	−0.258	−0.121 to −0.024	−2.940	0.004*
Preoperative HKA angle deviation	0.166	0.411	0.100–0.232	4.962	0.000*
Operation time	−0.001	−0.011	−0.009 to 0.008	−0.123	0.902
Preoperative VAS	0.155	0.166	−0.011 to 0.322	1.847	0.067
Preoperative KSS	−0.027	−0.236	−0.047 to −0.007	−2.672	0.009*
Interventions	1.370	0.331	0.667–2.073	3.859	0.000*
Preoperative ROM	−0.022	−0.217	−0.040 to −0.004	−2.441	0.016*

KSS, Knee Society score; ROM, range of motion; VAS, 10-cm visual analog scale.

* *P<*0.05.

Subsequently, preoperative HKA angle, preoperative HKA angle deviation, preoperative KSS, interventions, and preoperative ROM were included in multiple linear regression analysis. After statistical analysis, a statistical significance was observed with respect to interventions (*P*=0.002) and preoperative HKA angle deviation (*P*=0.003) (Table 3). According to the results of multiple linear regression analysis, when the preoperative HKA angle deviation increased by 1°, the postoperative HKA angle deviation increased by 0.134° (*β*=0.134; 95% CI: 0.045–0.222; *P*=0.003). When the interventions changed from robotic‑assisted system to CONs, the postoperative HKA angle deviation increased by 1.060° (*β*=1.060; 95% CI: 0.401–1.718; *P*=0.002). No statistical correlation was found between other factors and postoperative HKA angle deviation (*P*>0.05).

**Table 3:** Results of multiple linear regression analysis

Variables	*β*	*β* ate	95%CI	*t*	*P* value
Preoperative HKA angle	0.007	0.025	−0.053 to 0.067	0.231	0.818
Preoperative HKA angle deviation	0.134	0.332	0.045–0.222	2.997	0.003*
Interventions	1.060	0.256	0.401–1.718	3.184	0.002*
Preoperative ROM	−0.010	−0.100	−0.027 to 0.006	−1.216	0.226
Preoperative KSS	−0.018	−0.157	−0.036 to 0.000	−1.961	0.052

KSS, Knee Society score; ROM, range of motion.

* *P<*0.05.

Based on the results of multiple linear regression analysis, patients were divided into slight lower extremity alignment deviation group (preoperative HKA angle deviation <6°) and severe lower extremity alignment deviation group (preoperative HKA angle deviation ≥6°). The follow-up imaging parameters and clinical outcomes were then analyzed^19^.

### Results of patients with slight preoperative lower extremity alignment deviation

For patients with slight preoperative lower extremity alignment deviation, the postoperative HKA angle deviation was 1.56±0.95° in the RAS group and 2.25±1.54° in the CON group. The CFCA deviation was 0.79±0.62° in the RAS group and 0.99±0.73° in the CON group. The CTCA angle deviation was 0.90 (1.90)° in the RAS group and 1.80 (0.92)° in the CON group. The SFCA angle deviation was 1.20 (2.20)° in the RAS group and 1.20 (1.63)° in the CON group. The STCA angle deviation was 1.98±1.60° in the RAS group and 1.92±1.34° in the CON group. There was no significant difference in angle deviation between the two groups (*P*>0.05) (Table 4).

**Table 4:** Individual component positions and lower limb alignment in patients with slight preoperative lower extremity alignment deviation

		Angle deviation from neutral angle				
Group	*n*	STCA	SFCA	CTCA	CFCA	HKA
RAS	23	1.98±1.60	1.20 [2.20]	0.90 [1.90]	0.79±0.62	1.56±0.95
CON	14	1.92±1.34	1.20 [1.63]	1.80 [0.92]	0.99±0.73	2.25±1.54
*t*/*Z*		0.119	−0.251	−1.521	−0.876	−1.510
*P* value		0.906	0.802	0.128	0.387	0.147

CFCA, coronal femoral component angle; CON, conventional techniques; CTCA, coronal tibial component angle; HKA, hip–knee–ankle; RAS, robotic‑assisted system; SFCA, sagittal femoral component angle; STCA, sagittal tibial component angle.

There was no significant difference in the rate of outliers for SFCA, STCA, CTCA, and CFCA between the two groups (*P*>0.05), while the rates of the postoperative HKA angle outliers in the RAS and CON groups were 0.0 and 28.6%, respectively, and the difference was statistically significant (*P*=0.007) (Table 5).

**Table 5:** Outliers for component positions and lower limb alignment among patients with slight preoperative lower extremity alignment deviation

	Percentage of knees with implant aligned outside±3° from neutral angle				
Group	STCA	SFCA	CTCA	CFCA	HKA
RAS (%)	4.3	17.4	4.3	0.0	0.0
CON (%)	14.3	0.0	0.0	0.0	28.6
*χ* ^2^	1.154	2.730	0.626	-	7.368
*P* value	0.283	0.098	0.429	1.000	0.007*

CFCA, coronal femoral component angle; CON, conventional techniques; CTCA, coronal tibial component angle; HKA, hip–knee–ankle; RAS, robotic‑assisted system; SFCA, sagittal femoral component angle; STCA, sagittal tibial component angle.

* *P<*0.05.

There was no significant difference in postoperative KSS, postoperative VAS score, and postoperative ROM between the two groups (*P*>0.05), but the operation duration was 155.70±45.62 min in the RAS group and 107.50±28.41 min in the CON group; this difference was statistically significant (*P*<0.001) (Table 6).

**Table 6:** Postoperative data at 12 weeks for patients with slight preoperative lower extremity alignment deviation

Group	*n*	KSS	VAS	ROM	Operation time
RAS	23	60.35±14.59	3.56±2.39	109.57±15.29	155.70±45.62
CON	14	56.86±15.41	3.41±2.05	116.64±10.89	107.50±28.41
*t*		0.691	0.185	−1.511	3.960
*P* value		0.494	0.855	0.140	0.000*

CON, conventional techniques; KSS, Knee Society score; RAS, robotic‑assisted system; ROM, range of motion; VAS, 10-cm visual analog scale.

* *P<*0.05.

### Results of patients with severe preoperative lower extremity alignment deviation

Among patients with severe preoperative lower extremity alignment deviation, the postoperative HKA angle deviation was 2.09±1.18° in the RAS group and 3.57±2.73°in the CON group; the difference was statistically significant (*P*=0.001). The CFCA deviation was 1.16±0.78° in the RAS group and 2.13±2.04° in the CON group (*P*=0.004). The CTCA angle deviation was 1.32±0.90° in the RAS group and 1.55±1.29° in the CON group. The SFCA angle deviation was 1.50 (1.30)° in RAS group and 1.80 (1.70)° in the CON group. The STCA angle deviation was 2.00±1.66° in the RAS group and 2.22±1.84° in the CON group. There was no significant difference in CTCA, SFCA, and STCA angle deviation between the two groups (*P*>0.05) (Table 7).

**Table 7:** Individual component positions and lower limb alignment in patients with severe preoperative lower extremity alignment deviation

		Angle deviation from neutral angle				
Group	*n*	STCA	SFCA	CTCA	CFCA	HKA
RAS	39	2.00±1.66	1.50 [1.30]	1.32±0.90	1.16±0.78	2.09±1.18
CON	47	2.22±1.84	1.80 [1.70]	1.55±1.29	2.13±2.04	3.57±2.73
*t*/*Z*		−0.579	−0.686	−0.947	−2.992	−3.347
*P* value		0.564	0.493	0.347	0.004*	0.001*

CFCA, coronal femoral component angle; CON, conventional techniques; CTCA, coronal tibial component angle; HKA, hip–knee–ankle; RAS, robotic‑assisted system; SFCA, sagittal femoral component angle; STCA, sagittal tibial component angle.

* *P<*0.05.

There was no significant difference in the rate of outliers for SFCA, STCA, and CTCA between groups. The rates of the postoperative HKA angle outlier in the RAS group and CON group were 5.1 and 44.7%, respectively, and the difference was statistically significant (*P*<0.001). The rates of outliers for CFCA in the RAS group and CON group were 0.0 and 21.3%, respectively, and the difference was statistically significant (*P*<0.05) (Table 8).

**Table 8:** Outliers for component positions and lower limb alignment among patients with severe preoperative lower extremity alignment deviation

	Percentage of knees with implant aligned outside±3° from neutral angle				
Group	STCA	SFCA	CTCA	CFCA	HKA
RAS (%)	12.8	7.7	0.0	0.0	5.1
CON (%)	19.1	21.3	6.4	21.3	44.7
*χ* ^2^	0.626	3.065	2.579	9.390	17.019
*P* value	0.429	0.080	0.108	0.002*	0.000*

CFCA, coronal femoral component angle; CON, conventional techniques; CTCA, coronal tibial component angle; HKA, hip–knee–ankle; RAS, robotic‑assisted system; SFCA, sagittal femoral component angle; STCA, sagittal tibial component angle.

* *P<*0.05.

There was no significant difference in postoperative KSS, postoperative VAS score, and postoperative ROM between the two groups (*P*>0.05), but the operation duration was 158.56±46.32 min in the RAS group and 124.74±40.97 min in the CON group, and the difference was statistically significant (*P*<0.001) (Table 9).

**Table 9:** Postoperative data at 12 weeks for patients with severe preoperative lower extremity alignment deviation.

Group	*n*	KSS	VAS	ROM	Operation time
RAS	39	60.44±14.39	2.89±2.13	115.28±14.57	158.56±46.32
CON	47	58.40±18.50	2.91±2.09	114.72±25.43	124.74±40.97
*t*		0.559	−0.042	0.122	3.591
*P* value		0.577	0.967	0.904	0.001*

CON, conventional techniques; KSS, Knee Society score; RAS, robotic‑assisted system; ROM, range of motion; VAS, 10-cm visual analog scale.

* *P<*0.05.

## Discussion

Robotic-assisted knee replacement is the hotspot in the field of total knee replacement. Theoretically, robotic-assisted knee replacement can result in better lower limb alignment. However, it is still controversial whether there is an advantage with regard to alignment improvement with robotic-assisted knee replacement compared with conventional knee replacement^6^–^9^. But at the same time, it also prolongs the operation time, adds additional tests and costs, and there may be complications related to the robot-assisted system, so whether patients can benefit in the long term is controversial^20^,^21^. It has been reported that surgeons require a considerable level of training on an RAS to optimize safety and reliability^22^. In this prospective, multicenter, randomized controlled trial, we found that preoperative HKA angle deviation was a risk factor for postoperative HKA angle deviation. In patients with preoperative HKA angle deviation less than 6°, there was no significant difference between the robotic-assisted and conventional strategies in terms of postoperative HKA angle deviation. However, when 180±3° was used as the standard, the rate of the postoperative HKA angle outliers in the RAS group was significantly better than that in the CON group (*P*<0.05). In patients with preoperative HKA angle deviation more than or equal to 6°, the rates of outliers for postoperative HKA angle and CFCA in the RAS group were better than those in the CON group, and the HKA angle deviation and CFCA deviation were significantly lower in the RAS group than in the CON group (*P*<0.05). It is worth noting that the operation duration in the RAS group was significantly longer than that in the CON group (*P*<0.05). There was no significant difference in other indices between the two groups (*P*>0.05). At the same time, the centers included in this study were one of the best joint surgery centers in China, which may have influenced the results of the study.

Improved lower limb alignment and component positioning in TKA are important factors to ensure satisfactory postoperative function, patient satisfaction, and prosthesis survival^23^,^24^. Although there is no clear standard for limb axis and component positioning after TKA so far, most surgeons believe that it is appropriate to restore the lower limb alignment to neutral^25^. According to published reports, when the prosthesis position and lower limb alignment are deviated more than 3° from neutral, the failure rate and revision rate of TKA surgery are significantly increased^17^,^26^. In conventional TKA, an intramedullary guide system is used for the distal femoral cut and extramedullary guide system for proximal tibial cut, which are highly dependent on the operator’s experience, and ~10–20% of patients who undergo TKA following this technique are not satisfied with the results of the operation; this may be related to prosthesis misalignment and poor lower limb alignment^9^. To deal with this situation, RAS is used in TKA surgery, which aims to improve the surgical and clinical outcomes of TKA, and the number of surgeons using this technology is gradually increasing in recent years^6^. Some studies have shown that RAS can help improve mechanical axis of the leg and component positioning better than CON^9^,^27^,^28^. In addition, the robot-assisted system not only obtains more appropriate soft tissue balance but also protects soft tissue more effectively^29^,^30^.

To date, many studies comparing RAS and CON with regard to the effectiveness of improving prosthesis positions and lower limb alignment and postoperative function have been reported, but the conclusions are contradictory. Khlopas *et al*.^30^ and Marchand *et al.* 31 show that RAS can achieve better ROM and knee joint function than CON. However, Li et al found that there was no significant difference in postoperative ROM and knee functional score between RAS and CON, which was consistent with the findings of our this studies^12^. A large number of research results show that the RAS can effectively help doctors restore the mechanical axis to 180° and improve prosthesis alignment, thus reducing the failure rate and revision rate of TKA^32^–^34^. Our results showed that there was no significant difference in knee functional score and ROM after TKA between patients with preoperative HKA angle deviation less than 6°and those with HKA angle deviation greater than or equal to 6°. It has been previously reported that compared with CON, RAS cannot effectively improve the position of knee components^35^. In this study, only patients with preoperative HKA angle greater than or equal to 6° had intergroup significant differences in the rate of outliers of CFCA and CFCA deviation. It is suggested that a robotic-assisted TKA system has certain advantages in adjusting the prosthesis position for patients with severe preoperative lower extremity alignment deviations (preoperative HKA angle deviation greater than or equal to 6°). Although the rate of outliers for postoperative HKA angle in patients with preoperative HKA less than 6° was significantly different from that in patients with preoperative HKA angle less than 6°, the difference may be related to projection error in radiographic films^36^. In addition, this phenomenon may be associated with the small sample size of our study, the fact that all doctors included in the study were experienced doctors, and/or robotic-assisted system-related errors. Our most important finding is that RAS is not superior to CON for the patients with slight preoperative lower extremity alignment deviations (preoperative HKA angle deviation less than 6°). This is similar to the results of previous studies, reporting that RAS was not more effective than CON in restoring the mechanical axis of the leg at the last follow-up^10^,^35^. Although not statistically significant, the results were similar to the present study in that RAS achieved an HKA angle closer to the normal axis^10^,^35^. For patients with severe preoperative lower extremity alignment deviations, RAS can restore the mechanical axis of the leg to the neutral angle more effectively than CON. Bae *et al*.^37^ found that preoperative varus deformity affects postoperative prosthesis alignment and suggested the need for strategies to ensure accurate alignment.

In this study, by the means of single-factor and multifactor linear regression analysis, it was found that the preoperative HKA angle deviation would affect the recovery of the postoperative HKA angle deviation (*β*=0.134; 95% CI: 0.045–0.222). The results also verified that RAS was more effective than CON for patients with severe preoperative lower extremity alignment deviations (preoperative HKA angle deviation greater than or equal to 6). At present, there is a lack of follow-up studies on the long-term survival rate, knee functional score, and ROM with respect to RAS for TKA. Li *et al.* 12 confirmed that RAS does not achieve better clinical outcomes than CON in short-term, and it is not clear whether RAS can achieve better clinical and radiological outcomes in the medium and long term.

This study still has many limitations. First, we cannot eliminate the impact of the small sample size in this study on the results. Second, this study only made a short-term follow-up observation of patients, and the long-term effect of RAS remains unclear. Third, the bias that exists in this study is that in the RAS group, because surgeons invested a lot of time and cost, they will inevitably operate more carefully in the surgery, so this factor may have influenced the results of the study. Finally, the centers included in this study were centers where a large number of knee replacements are performed in China, and all investigators were prominent Chinese joint surgeons; the skill level of the surgeons could have influenced the results.

## Conclusion

In conclusion, robotic-assisted TKA system in this study is a safe and effective system for TKA. Compared with CON, RAS cannot obtain better clinical and radiographic outcomes for patients with slight preoperative lower extremity alignment deviations; for patients with severe preoperative lower extremity alignment deviations, RAS can help achieve better alignment of the limb axis and prosthesis alignment, but there is no significant difference in clinical outcomes between the two groups. Moreover, we found that preoperative HKA angle deviation affects the postoperative mechanical axis of the leg. We suggest that RAS should be routinely used in patients with severe preoperative lower extremity alignment deviations. Whether RAS has better long-term radiographic and clinical outcomes than traditional surgical techniques needs to be further studied.

## Ethical approval

This study was approved by the ethics committee of the second affiliated Hospital of Xi’an Jiaotong University (Permit Number: XJTU 2021-015) and was carried out in accordance with the Declaration of Helsinki of the World Medical Association. All enrolled patients provided written informed consent. The study was registered with chictr.org.cn/enindex.aspx.

## Sources of funding

Clinical Application-Oriented Medical Innovation Foundation from National Clinical Research Center for Orthopedics, Sports Medicine and Rehabilitation (2021-NCRC-CXJJ-ZH-21).

## Authors’ contribution

R.T., K.W., P.Y., S.Y., J.W., Z.S: completed patient recruitment and surgery. J.L. trained the robot operators on robot operation before the clinical trial was conducted. X.L. and N.K.: measured the patient’s imaging data. R.T.: collected research data. X.D. analyzed the clinical trial data. R.T. and X.D. authored the manuscript.

## Conflicts of interest disclosure

The authors declare that they have no financial conflict of interest with regard to the content of this report.

## Research registration unique identifying number (UIN)

None.

## Guarantor

Run Tian.

## Provenance and peer review

Not commissioned, externally peer-reviewed.

## Data availability

The data are not publicly available, as participants in this study need their data for further analysis.
